# Individualized mRNA Vaccines in Melanoma—Where Do We Stand?

**DOI:** 10.3390/vaccines13090986

**Published:** 2025-09-21

**Authors:** Ioanna Gazouli, Dimitrios Bafaloukos, Christos Koutserimpas, George Samonis

**Affiliations:** 1 Medical Oncology Department, Metropolitan Hospital, Neon Faliron, 18547 Athens, Greece; ioannagazouli@gmail.com (I.G.); dimmp@otenet.gr (D.B.); 2 School of Rehabilitation Health Sciences, Rio Campus, University of Patras, 26504 Patras, Greece; koutserimpas@upatras.gr; 3 Department of Medicine, University of Crete, 71500 Heraklion, Greece

**Keywords:** mRNA vaccine, individualized neoantigen treatment, lipid nanoparticles, immunotherapy, melanoma, antigen presentation

## Abstract

Immunotherapy, consisting mainly of immune checkpoint inhibitors, has been successfully employed in the treatment of early and advanced-stage melanoma for more than ten years. Personalized mRNA vaccines represent the next evolutionary step, offering patients a treatment unique to them and their tumor, whilst putting recent, significant technological and scientific advances into practice. Clinical and preclinical data about mRNA vaccines are now emerging, further encouraging research and spreading enthusiasm among patients and physicians. Nonetheless, a lot remains to be discovered about mRNA vaccines’ mechanisms of action, their actual effect on the immune cells of the patient, and successful mRNA delivery to the host.

## 1. Introduction

Cutaneous melanoma represents 1.7% of malignancies diagnosed worldwide, its annual incidence steadily rising from 20 to 23.5 new cases per 100,000 people during the decade 2012–2022 [1,2,3,4]. Notably, men seem to be increasingly affected, with a melanoma incidence of almost 30 cases per 100,000 people, within the year 2022 [1]. Although the mean age of diagnosis is estimated to be 59 years, about six to seven individuals younger than 50 years are diagnosed with melanoma every year since 2013 [1]. Melanoma of the skin is now emerging as a major public health issue [1,2,3,4].

Melanoma derives from melanocytes, melanin-pigmented cells located in the dermis, meant to absorb harmful ultraviolet radiation [5,6]. Nonetheless, mutated melanocytes may give rise to a potentially life-threatening malignancy [5,6,7]. In fact, melanomas might be divided into two categories, depending on the primary location on either chronically sun-damaged (CSD) or non-CSD skin [7,8]. CSD melanomas are associated with prior long-term sun exposure and older patient age, while non-CSD melanomas tend to appear in younger individuals, located on non-exposed areas such as the trunk [7,8]. Acral melanomas, presenting in palms, soles of feet, and nails, are considered to form another category, not being associated with sun exposure, and often develop at a slow pace, which makes them easy to overlook unless dermoscopy is performed [8,9].

Indeed, melanocytes may develop mutations that make them able to overmultiply, escape immune surveillance, and translocate to other departments of the host’s tissues, while inactivating crucial cell death pathways [5,6,7,8]. In fact, more than 60% of cutaneous melanomas host the BRAFV600E oncogenic mutation; the substitution of the amino acid valine by glutamic acid, at position 600, results in a constantly active state of the BRAF kinase, leading to incessant activation of the MAP kinases pathway, enhancing proliferation and survival of the malignant melanocytes [5,6,7,8,9,10,11]. Nonetheless, BRAFV600E mutation nowadays represents a valuable therapeutic target, as powerful regimens of combined BRAFV600E and MEK kinase inhibitors have been put into practice [12,13,14]. According to the most recent follow up of the Columbus trial [13], assessing the newest combination, encorafenib/binimetinib, the overall response rate among metastatic melanoma patients exceeds 60%, with median disease-specific survival reaching 3 years since treatment initiation.

Nevertheless, not all melanomas harbor BRAF V600 targetable mutations. In this case, immunotherapy with immune checkpoint inhibitors offers a highly effective therapeutic strategy, widely applied since the introduction of ipilimumab in 2011 [15,16]. This monoclonal antibody [15,16], targeting Cytotoxic T-lymphocyte-associated protein 4, has been initially assessed as monotherapy, at doses as high as 10mg/kg, inducing response rates of 11% with high rates of concomitant toxicity. Currently, ipilimumab is administered at a lower dose, together with the anti-programmed cell death antibody, nivolumab, against metastatic melanoma [17], resulting in response rates as high as 58% and median disease-specific survival exceeding 10 years since the combined regimen was initiated.

Meanwhile, more immune checkpoint inhibitors are emerging. Ten-year follow-up trial data have now also become available for the anti-PD-1 agent pembrolizumab, showing overall survival rates of 34% among metastatic melanoma patients [18] while novel combinations such as nivolumab/relatlimab keep emerging [19], broadening treatment options. Great progress has also been noted in the perioperative treatment setting; adjuvant immunotherapy administration is able to prevent disease relapse after resection of melanomas extending deeply within the dermis and/or presenting with lymph node dissemination [20,21,22]. In case of clinically apparent, infiltrated lymph nodes, patients may also benefit from neoadjuvant treatment, which seems to reduce the need for extensive surgery and postoperative treatment, in patients achieving major pathologoanatomic response [23,24,25].

However, resistance eventually develops, as malignant melanocytes find ways to escape the host’s immune system, adjusting intracellular pathways, altering expressed antigens, and interfering with antigen presentation and cytokine signaling [26,27]. It could also be argued that immune checkpoint inhibitors, despite having revolutionized melanoma treatment, act basically as immune cell enhancers by impeding cytotoxic T cell receptors, such as PD-1 and CTLA-4, which mediate T lymphocyte inactivation and/or exhaustion [28,29,30]. In other words, immune checkpoint inhibitors may build up a hypervigilant immune system, inevitably inducing autoimmune toxicities. However, these agents miss out on the opportunity to sharpen the effector cells’ activity to identify malignant cells in a sensitive and more specific manner [28,29,30].

It now seems possible that the above-mentioned shortcomings of the established immunotherapy regimens might be resolved by personalized mRNA vaccines, regarded as the upcoming breakthrough in melanoma treatment [31]. This novel approach aims to optimize the host’s immune system performance by exposing tumor-specific proteins to lymphocytes. Highly efficient mRNA delivery by lipid nanoparticles (LNPs) together with genetic analysis allowing recognition of cancer proteins unique to every patient may both be regarded as the cornerstones of individualized neoantigen mRNA vaccines [31,32,33]. These tremendous scientific and technological advances have enabled researchers to exploit the genetic and phenotypic changes in cancer as footprints to help the immune system identify tumor cells, producing a drug absolutely unique to every patient [31,32,33].

## 2. Opening a New Era in Melanoma

It cannot be doubted that the findings of the small yet substantial KEYNOTE-942 trial opened a new era in the treatment of melanoma [34,35]. The 157 participants, once having undergone complete excision of high-risk cutaneous melanoma, stage IIIB to IV, were randomly assigned to receive standard adjuvant treatment with pembrolizumab at 200mg every 21 days for up to one year, with or without personalized mRNA vaccine (i.e., V940), given intramuscularly for up to nine administrations in total. Most of the recruited patients had clinically detectable, disseminated regional lymph nodes, whilst 15% of them had resected oligometastatic disease [34,35].

The study achieved its primary endpoint, as V940 addition was found to significantly reduce disease recurrence by 44%, according to the most recent update [9,10]. Notably, a statistically significant recurrence-free survival (RFS) difference was observed regardless of positive or negative PD-L1 status, defined by a 1% cut off. In fact, patients with PD-L1 expression lower than 1% seemed to retrieve even more evident clinical benefit from V940 administration, compared to patients with detectable PD-L1 [34,35]. At 18 months since treatment initiation, 79% of patients under pembrolizumab plus V940 were still free of metastatic disease, compared to 62% under single-agent pembrolizumab. As it would be expected, further follow up will be required for any overall survival benefit to be established [34,35].

The exciting findings mentioned above [34,35] are now being assessed in a larger scope, in the context of the ongoing international, double-blind, placebo-controlled randomized trial INTerpath-001 (i.e., V940-001) [36]. This latter study follows the pattern of KEYNOTE-942, slightly altering the treatment regimen; pembrolizumab is given at nine doses of 400 mg every six weeks, the intramuscular injection of V940 or placebo being administered in 21-day cycles. Patient inclusion criteria have been extended to allow recruitment of patients with stage IIB and IIC melanoma, cutaneous or acral [36]. The primary endpoint is once again RFS, secondary endpoints being OS, DMFS, safety, and quality of life (QoL). More than a thousand patients have received treatment, and study outcomes are eagerly anticipated [37].

Given the effectiveness of the pembrolizumab/mRNA vaccine combination in the adjuvant setting, oncologists are now wondering if it can improve outcomes in patients with metastatic disease. The upcoming multicenter V940-012/INTerpath-012 trial [38] is already open to recruitment in nine countries, aiming to enroll about 160 patients, in order to determine if the addition of an mRNA vaccine to the standard first-line pembrolizumab could be of any benefit to inoperable melanoma patients. Progression-free survival (PFS) will be the primary endpoint. Should V940-012 turn out positive, a valid counterweight of the powerful ipilimumab/nivolumab combination might become available. Regardless, it cannot be doubted that personalized mRNA vaccines have already inaugurated a new chapter in cancer treatment.

The safety and tolerability of the personalized mRNA-4157 vaccine had been initially assessed in the context of a phase I study, KEYNOTE-603 (NCT03313778) [39,40,41]. Seventy-nine patients with miscellaneous neoplasms, including colorectal cancer, melanoma, and cervical cancer, were recruited to receive mRNA-4157, with sixteen of them receiving it as single-agent treatment in the adjuvant setting, and sixty-three of them undergoing systematic treatment for unresectable disease, with both mRNA vaccine and pembrolizumab. Once again, mRNA-4157 safety was confirmed, as only mild toxicities occurred. In the combination, systematic treatment cohort, eight partial and three complete responses were noted [40]. Among the adjuvant cohort patients, 13 managed to complete the vaccination schedule, with 11 of them still disease free at 72 weeks on study [40].

At the moment, there are nine active trials assessing the efficacy of mRNA-4157, with about six of them still being open to recruitment [42]. In most of them, the vaccine is administered in combination with pembrolizumab, whereas in V940-011/(INTerpath-011), it is combined with intravesical BCG, aiming to prevent high-risk, non-muscle invasive bladder urothelial cancer from recurrence. In the majority of cases, the vaccine is assessed in the adjuvant setting, against melanoma (in two trials), renal cell carcinoma (in one trial), and cutaneous squamous cell carcinoma (in one trial). In the recently initiated V940-012, it is used against unresectable melanomas, together with pembrolizumab, and compared head-to-head with single-agent pembrolizumab (see Table 1) [42].

Still, clinical trial data regarding personalized mRNA vaccination remain scarce [39,40,41,42]. However, as the vaccines are being increasingly assessed in international clinical trials, researchers are seeking more abundant and valid clinical data, in terms of both tolerability and efficacy [43]. Meanwhile, preclinical and translational research is also evolving, aiming to dissect the mechanism of action of personalized vaccines and their effect on the host’s immunity against malignancies [41].

## 3. Mechanism of Action

Personalized mRNA vaccines are designed to inform the immune system of the host about the characteristic proteins expressed by melanoma or any other tumor cells, so that the former can successfully identify and destroy the latter [32]. This is performed by an ex vivo-created mRNA sequence, which encodes for characteristic, highly immunogenic tumor antigens, which is delivered to the host [32,33].

**Figure 1:**
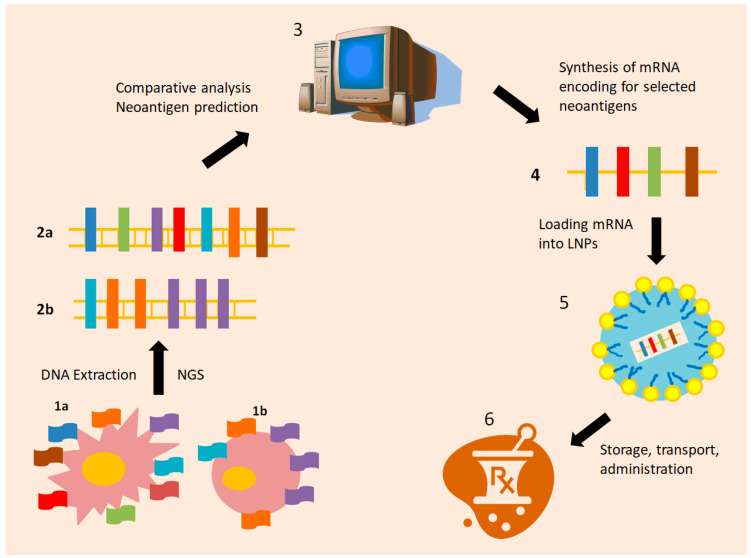
Manufacturing individualized mRNA vaccines: a Schematic representation. Malignant cells exhibit multiple neoantigens, resulting from various genetic and epigenetic alterations (1a). Normal cells share a common, repetitive variety of normal antigens (1b). DNA from cancer tissue and blood samples for each patient is analyzed by Next-Generation Sequencing. Once cancer (2a) and normal cell DNA (2b) are sequenced, the resulting data sets are further examined in parallel by computational systems (3). Highly immunogenic neoantigens are selected, and an mRNA sequence encoding for them is created ex vivo (4) and enclosed in Lipid nanoparticles (LNPs) for protection and stability (5). In this form (6), the individualized mRNA vaccine, encoding for significant tumor-specific neoantigens, may be stored, transferred, and administered to the patient. Each such individualized vaccine is uniquely designed for only one patient.

Vaccine mRNA-4157, being assessed in multiple trials at the moment, as already described above, might be considered as a personalized vaccine prototype [32,33,39,40,41,42,43,44]. It is designed based on the tumor-specific antigens that distinguish the cancer cells from the normal ones and aspires to encode for the neoplastic antigens that could motivate immune cells against the tumor. Neoplastic tissue and blood samples of the patient are analyzed by next-generation sequencing, and genetic sequence data sets are compared by state-of-the-art computational systems [32,33,39,40,41,42,43,44]. Once genetic suspicious loci, potentially representing malignant shifts, are identified, the ones amongst them with the highest immune-enhancing potential are selected [44]. An mRNA molecule, encoding for these neoantigens, regarded as unique both to tumor and host, i.e., individualized, is ex vivo produced and then safely encapsulated within lipid nanoparticles. In this form, it may be refrigerated, stocked, and transferred, let alone easily administered to the patient by a simple intramuscular injection [39,40,41,42,43,44] (see Figure 1).

**Figure 2:**
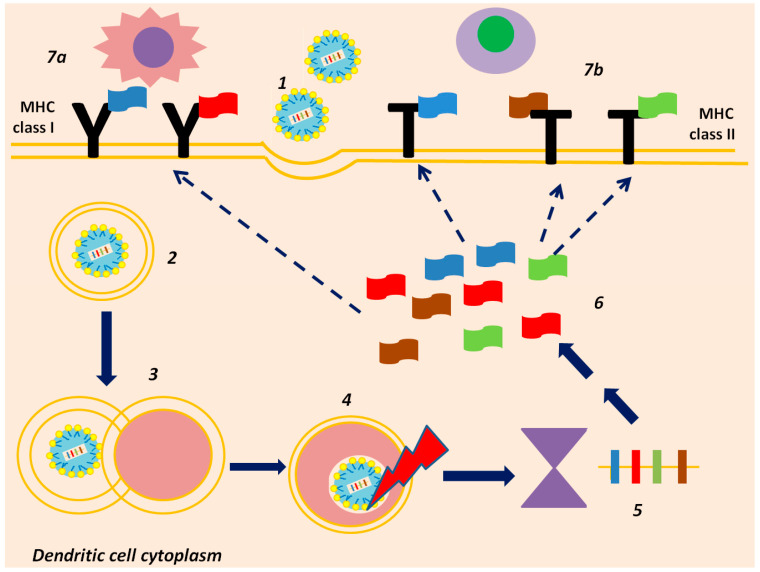
Schematic representation of how mRNA neoantigen vaccines enhance immunity against cancer cells. Once the LNP-enclosed mRNA molecule is endocytosed by the dendritic cells (1), an endosome is formed (2). The endosome may fuse with a lysosome (3), forming an endolysosome that has to be disrupted in order for the encoding mRNA to be released intact into the cytoplasm (endosomal escape, 4). mRNA is then translated by ribosomes (5) into neoantigens representing the phenotypic profile of cancer cells (6). Neoantigens may then be linked to MHCs, class I (7a) and II (7b), eventually being exposed to both cytotoxic CD8+ and helper CD4+ T-lymphocytes, respectively.

Once the vaccine is administered to the host, it is incorporated by the antigen-presenting cells, and it is translated into tumor proteins and exposed on the antigen-presenting cell surface, thus becoming available to lymphocytes [43,44,45] (see Figure 2). Subsequently, post-vaccination T lymphocytes may be more prone to activate, expand, and create highly specialized clones, once they encounter tumor cells, optimizing immunity against cancer (see Figure 2). Combination with an immune checkpoint inhibitor is meant to boost T cell activity, while preventing T cell exhaustion [45,46].

All in all, the vaccine and classic immunotherapy combination aspires to a stronger, but also more intelligent, cellular immunity against tumor cells [32,33,39,40,41,42,43,44]. Undoubtedly, basic principles of mRNA vaccine treatment against cancer have now long been established [39,40,41,42,43,44]; however, putting such a concept into practice only became feasible after decades of scientific and technological advances [39,40,41,42,43,44,45,46].

## 4. Old Ideas Under a New Light: Targeting the Dendritic Cell

The dendritic cell (DC) is regarded as the ultimate professional antigen-presenting cell; it could be argued that it orchestrates the whole immune reaction cycle [47]. Taking its name from its characteristic tree-like appearance, it is a macrophage able to move with agility throughout the host’s system, thanks to its cytoplasmic branches, and it is also able to engulf and digest all kinds of molecules, from innate, normal proteins to viral, bacterial, and neoplastic antigens [48,49]. Once endocytosed by the DC, proteins and antigens are decomposed, and their critical pieces, known as antigen epitopes, are exposed at the dendritic-cell surface, steadily bound to major histocompatibility complexes (MHCs), otherwise referred to as human leukocyte antigens (HLA) [50].

The inherent asset of dendritic cells, making them particularly efficient in antigen presentation, is that they have both MHC class I and II, which mediate interaction with both CD8+ positive, cytotoxic T lymphocytes, and CD4+-expressing helper T lymphocytes, respectively [51,52,53]. This means they directly activate CD8+ T lymphocytes, which eliminate malignant cells, but they do not miss at activating helper cells, too [23,24]. The latter further enhances cytotoxicity and immune cell memory, secretes cytokines, and also enhances B lymphocytes’ activity against cancer [54,55].

As mentioned above, translocation is another powerful asset of DCs [54,55,56]. In contrast to the regular tissue macrophages, which, once they have differentiated into their final, functional form, cannot relocate, a DC, loaded with cancer antigens, may travel through the lymph system and reach the lymph nodes [56]. There, it can provoke naïve, inactive lymphocytes to multiply, mature, and enter the host’s lymphovascular system, where they are able to track down and destroy cancer cells, a phenomenon described as lymphocyte priming [57].

The ability of DCs to move within the skin and lymph nodes of melanoma patients has been successfully captured in vivo as early as 2005 [27]. The researchers transfected human dendritic cells with superparamagnetic iron oxide, injected them into melanoma patients, and tracked their movement by the use of magnetic resonance imaging. Indeed, dendritic cells were able to advance from their intradermal injection site to the proximal lymph node basin and also translocate among different lymph node stations [58].

The idea of targeting a cell that is fundamental to the immune reaction is not new [59]. Mainly, three types of vaccines, all aiming to empower DC activity, have been employed. First, peptide antigens frequently encountered in certain tumor types, such as Melan-A, gp100 peptide, and HMB-45 in melanoma, were isolated and injected into patients, so that they might be absorbed by DCs, which would, in turn, set off the lymphocytic immune response. However, such vaccines never made it to routine clinical practice, due to their modest efficacy [60,61], especially compared to the highly effective immune checkpoint-inhibiting antibodies.

Second, the attempt to, in ex vivo, process the host’s monocytes, in order to inject them back into the patient as fully equipped DCs after incubation with growth factors and tumor proteins, did not prove to be particularly efficient [62]. In one comparative trial, published in 2012, patients with metastatic melanoma were treated with an autologous transplantation of DCs, which were ex vivo loaded with melanoma-associated antigens, and they managed to induce a 2-year survival rate of 72%, compared to 31% for the patients who underwent irradiated tumor cell administration [63].

Lastly, in a more modern version, human DCs have been transfected ex vivo with cancer mRNA, so that, when returned to the patient, they would have incorporated the algorithm for producing cancer antigens and would be exposed to effector lymphocytes [64].

Regardless of their solid physiological and immunological background, all the above ideas did not show fruitful results, whilst being impractical and impossible to perform outside specialized centers [60,61,62,63,64]. In this context, modern mRNA vaccines are designed to achieve easy and safe injection of the mRNA encoding for tumor antigens, directly into the system of the patient/host, while focusing on dissecting personalized, tumor-specific neoantigens, both tumor and patient specific [32,33,34]. While individualized mRNA vaccines encode for tumor- and host-specific antigens, BNT111 aims to induce expression of the four most frequent melanoma-associated antigens, NY-ESO-1, MAGE-A3, tyrosinase, and TPTE, again utilizing mRNA vaccine technology. All the above-mentioned approaches are summarized in Table 2 [65].

## 5. Dissecting the Footprints of Cancer: Individualized Neoantigen Treatment (INT)

mRNA vaccines, such as V940, may be more accurately referred to as individualized neoadjuvant treatment (INT) [66]. This is to say that the mRNA molecule contained in the vaccine encodes for tumor-specific neoantigens unique to every patient and their tumor. If an antigen is defined as any molecule or molecular fragment that, once bound to HLA proteins, is identifiable by T cell receptors (TCR), then neoantigens should qualify as molecules prone to trigger a T cell response, whilst being tumor-specific or even unique to every tumor and host [67]. Thus, maybe for the first time in the history of medicine, treatment individualization has reached the point where each and every patient is treated by a one-of-a-kind, perfectly tailored therapeutic agent. Indeed, such an advance as that could never be tangible without next-generation sequencing (NGS), combined with computer science [68,69].

The V940 vaccine administered in KEYNOTE-942 and V940-001 trials, as mentioned above, is predicted to encode for up to 34 neoantigens [34,35,36], depicting the phenotypic profile of any patient’s melanoma cells, and setting the paradigm of INT in practice. Building an individualized mRNA vaccine follows four main steps:

1. NGS of both neoplastic tissue and peripheral blood specimens of the patient: NGS has revolutionized cancer genomics during the last decades [70], allowing parallel analysis of large cancer and normal cell DNA sequences, in a fast and efficient manner. Cancer and normal genome sequences may then be compared by a computational system capable of spotting alterations, patterns, and motives, and that is highly suspicious of deriving from somatic mutations taking place within tumor cells [71,72]. In this way, antigens unique to each patient and host may be identified and used as a boost of innate immunity.

2. Selection of the most immunogenic neoantigens: The characteristic cancer neoantigens can be evaluated for their potential to form stable connections with HLA proteins and TCRs, in order to reassure optimal antigen presentation and T lymphocyte activation, respectively. Antigen structure and binding potential might be evaluated by mass spectrometry or in vitro interaction with HLA proteins [73,74]. Nowadays, such predictions might also be carried out in silico by using computational programs and artificial intelligence [75] in order to reassure that neoantigens more prone to trigger an immune response by the host will be chosen.

3. Ex vivo mRNA synthesis: An mRNA sequence encoding for the selected, highly immunogenic, both tumor and patient specific neoantigens, is generated ex vivo. The mRNA molecule, once in the host’s system, will be internalized by DCs, mediating expression of the targeted neoantigens DCs surface, i.e., neoantigen presentation [74,75].

4. Stabilization of the neoantigen encoding mRNA: mRNA can be encapsulated as cargo inside lipid nanoparticles (LNPs), protecting it from changes in temperature, pH, and lytic enzymes [76,77]. Then, the individualized mRNA vaccine can be administered to the host, implementing a highly specialized, accurate, and efficient immune response against cancer cells.

In this same context, the Masterkey-265 [78] trial administered an oncolytic herpes virus to patients with unresectable melanoma, in combination with pembrolizumab. The oncolytic virus was directly injected into accessible subcutaneous lesions, aiming not only to induce local shrinkage but, even more importantly, to expose tumor-characteristic antigens to the immune system of the host, inducing an abscopal effect. No matter its undeniably plausible scientific background, Masterkey-265 failed to show any benefit in either delaying disease progression or prolonging patient survival [78].

In that light, innovation of the novel mRNA vaccines consists of two elements [32,33]: (1) Making use of tumor-specific/patient-specific neoantigens, also prone to a steady interaction with the immune system; and (2) Efficient packaging and delivery of the mRNA molecule.

## 6. Next Generation Packaging: The Nanoparticles

Safe mRNA delivery has been proven to be a challenging task for a multitude of reasons. First, as a single-strand molecule, lacking the powerful hydrogen bonds between base pairs that confer DNA its unbreakable structure, it is extremely vulnerable to temperature, humidity, and pH fluctuations, and is prone to be degraded by the widely spread RNAases [79,80]. Second, it should be encapsulated by the targeted, dendritic cells, for the transfection to succeed. And, last but definitely not least, it should make it intact to the DC ribosomes, in order to be translated into tumor antigens, which will be presented on the dendritic cell surface, eventually becoming available to lymphocytes. Liposomal nanoparticles may be the answer to all three challenges [81,82,83,84].

In previous experiments, scientists have attempted to bind protamine or lipid molecules to the mRNA as a stabilizer [81,82,83,84]. Liposomal nanoparticles, with a structure resembling to the cellular lipid membrane, seem now to provide a more effective solution, enclosing and reassuring the valuable mRNA cargo both from environmental conditions and RNAases. In this form, mRNA vaccines can be stored and maintained, also transported in long distances, allowing access to larger patient populations, even far from the laboratory where they have been constructed [81,82,83,84]. This feature makes modern mRNA vaccines much more practical, compared to approaches of the past, involving ex vivo dendritic cell transfection or live, attenuated virus injection.

Moreover, nanoparticles have been found to enhance endocytosis by dendritic cells as well. According to in vitro experiments, transfection of dendritic cell cultures by mRNA contained in lipid particles is more effective compared to alternative vectors, while nanoparticles with a higher concentration of lipids mediate higher rates of dendritic cell transfection [81,82,83,84].

There is one major setback, nonetheless: once endocytosed, the mRNA-loaded lipid nanoparticle still has to survive the endosome to lysosome fusion, a phenomenon described as endosomal escape [84,85,86]. The endosome containing the LNP vector might fuse with a lysosome, creating an endo lysosome that could result in decomposing the mRNA before it can be translated into protein [84,85,86]. It is not feasible to monitor lysosomal escape, and thus, there is no guaranteed method to enhance it. For all that is known, lipid concentration and transfection rate do not affect lysosomal escape or destruction; in vitro data indicate that even LNPs achieving high transfection rates may fail to induce protein expression by the transfected cell [84]. In these experimental structures, transfection failure is associated with a marked increase in lysosomes inside the target cell, further implying that mRNA in these cases underwent intracellular lysis, as any other endocytosized element would.

There are two notable theories about how lysosomal escape might occur, both indicating the importance of the structure and electric charge of the assessed LNP vectors [85,86,87]. First, the cone effect, taking place when the positively charged LNP surface interacts with the negative charge of the endo lysosome, altering its shape into a cone, which finally breaks, releasing the LNP intact [84]. Second, the proton sponge effect [84] is induced when the LNP provokes a hydrogen influx within the endo lysosome, which results in chloride and water influx as well, eventually causing the endo lysosome to deconstruct due to high internal osmotic pressure. At the moment, LNP science focuses on stereotactic structure, shape, and charge of the LNPs, aiming to increase transfection rates by creating vectors capable of surviving endosome–lysosome fusion [81,82,83,84,85,86].

mRNA vaccines, enclosed in LNP vectors, were first introduced during the COVID-19 pandemic, making effective vaccination feasible for a large group of people [88]. LNPs represent a promising technology already revolutionizing cellular transfection and mRNA delivery. All that being said, it is still important to further dissect how LNPs interact with human cells to optimize their performance [86,87].

Another remarkable aspect of LNP vectors is their potential ability to trigger inflammation [88]. It has been observed that LNP-mRNA vaccines administered against SARS-CoV-2 could provoke local inflammatory responses in up to 19% of patients, at the injection site, manifesting with pain, swelling, and redness, along with fever and systemic inflammatory responses [88,89]. Given that the mRNA molecule could not be recognized by T cell receptors, in order to directly initiate inflammatory reaction [87], it is highly probable that LNP vectors themselves may initiate immune reaction.

Notably, administration of LNP vectors containing cationic lipids in mice is associated with neutrophil infiltration of the injected sites, which would even lead to massive parenchymal lung inflammation when the LNPs were administered intranasally. In addition, systemic release of inflammatory cytokines, such as interleukin [IL]-1β and IL-6 was also observed [89,90].

In another interesting experiment [91], empty LNP vectors, containing SM-102, a cationic lipid also found in the anti-COVID-19 vaccine by Moderna, were administered to mice, inducing IL-6 and IL-1β secretion together with activation of the inflammasome pathway. Quite remarkably, mice may be able to better tolerate LNP vaccination, compared to humans, by initiating the expression of the anti-inflammatory cytokine IL-1 receptor antagonist (IL-1ra), thus reducing the cytokine-mediated toxicities [91]. Nevertheless, it is important to keep investigating discrepancies between human and murine immune reactions to LNPs, as such differences may impose significant safety implications [91].

In a more recent study, it has been found that the very endosomal escape, which is required for the mRNA antigen encoding sequence to make it to the ribosomal units and be translated into proteins, may induce endosomal membrane damage, creating pores that allow the entrance of proteins called galectins. Galectins are normally located in the cytosol, but once they have entered the endosome, they become attached to sugars of the inner endosomal membrane, initiating an inflammatory reaction [92]. Moreover, galectin inhibition has been found to mitigate LNP-induced inflammation. It is even more interesting that biodegradable, ionizable LNP vectors are able to escape endosomes whilst causing less damage to the endosomal membrane structure, thus decreasing galectin inflow and diminishing subsequent inflammatory response [92].

The inflammatory and immune-enhancing effects of LNPs remain to be thoroughly investigated in preclinical and translational studies, in order to optimize their use against cancer in humans. Understanding how LNPs may mediate local and systemic inflammation should allow scientists to develop more effective, but also more tolerable, mRNA vaccines, which will be more accurately tailored to the human immune system [90,91,92].

## 7. Proof of Concept: How mRNA Vaccination Affects Host Immunity

The LNP-packed mRNA individualized vaccines aspire to transfect DCs, turning them into intelligent agents of hosts’ immune systems. Clinical trial data suggest that their administration is of clinical benefit in real patients. Is there any evidence regarding the actual interplay between the vaccine, immune system, and cancer cells?

Analysis of a subgroup of patients with metastatic melanoma and non-small cell lung cancer receiving the personalized V940 vaccine, in the context of KEYNOTE-603 phase I trial [39,40,41]. Researchers examined patients’ T-lymphocytes pre- and post-administration of V940 by assessing their ability to secrete IFN-gamma and TNF, once exposed in vitro to cancer antigens. Indeed, after vaccine administration, hosts’ lymphocytes displayed an increased potential of secreting IFN-γ and TNF, with their ability intensifying with time in repeated assessments. Notably, certain neoantigen pools were more prone to provoke a lymphocytic response compared to others, further supporting that immunogenicity is a key feature of neoantigens that should be selected to be included in the mRNA sequence delivered to the patient. In addition, both CD4+ helper and CD8+ cytotoxic lymphocytes showed increased activation rates after V940 treatment [41].

Even more impressively, V940 administration seemed to initiate an expansion in surface HLA types of antigen-presenting cells of the patients [41]. A broader variety of HLA receptors is associated with an enhanced ability of presenting a larger spectrum of neoantigens, thus further sharpening the lymphocytes. This is consistent with the findings of a retrospective analysis of KEYNOTE-942 patients [32,33], where it was discovered that patients homozygous for HLA genetic loci, considered to have an innate defect in antigen presentation, were the most benefited by the V940 addition to their treatment.

## 8. Future Prospects and Points Requiring Further Clarification

**Figure 3:**
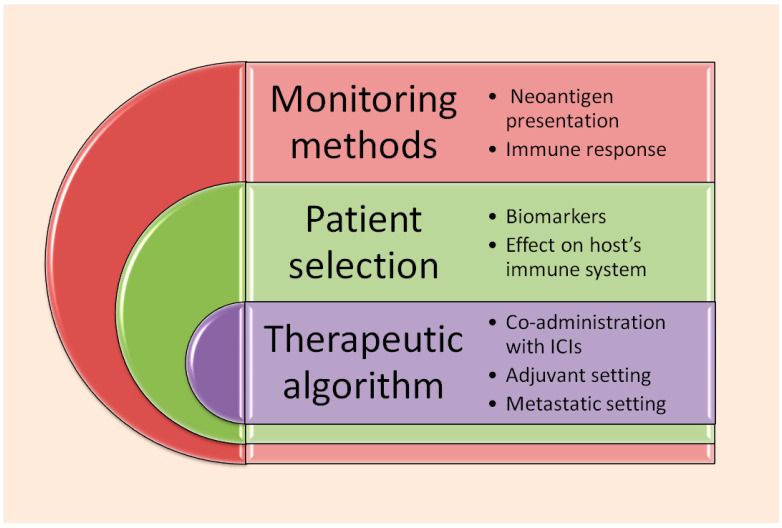
Future challenges regarding cancer mRNA individualized vaccines in melanoma and/or other solid malignancies. Future research might focus on three main categories: (1) Methods of assessment and in vivo monitoring of the actual effect of mRNA neoantigen vaccines on antigen presentation and subsequent cytotoxic immune response. (2) Establishment of patient selection criteria, so that treatment is administered to patients most likely to benefit, with good tolerance. (3) Planning a therapeutic algorithm containing individualized vaccines, among other available strategies in the adjuvant and metastatic treatment setting.

Nevertheless, the KEYNOTE-603 [41] findings are an indirect in vitro assessment of the vaccine’s ability to activate and ameliorate immune response against cancer. There is no method to monitor in vivo dendritic cell transfection, lysosomal escape of the vaccine, protein expression on DC surface, and T lymphocyte expansion and cytotoxic activity. Further studies, on both the clinical and preclinical levels are needed, in order to fully dissect the mechanism of action of mRNA vaccines and how it is affecting the host immune response (see Figure 3).

According to the latest data, V940 treatment does not seem to increase the rate of severe or immune-related adverse events, nor has it been found that it leads more patients to discontinue treatment [32,33]. However, longer follow up in the context of both clinical trials and routine practice is required to establish that individualized neoantigen treatment is a safe manner to increase patients’ benefit from classic, immune checkpoint-based immunotherapy.

Another problem consists of the hierarchy by which INT is going to be included in the therapeutic algorithm. Will it be employed in an adjuvant setting exclusively or is it going to become an essential part of first-line treatment against metastatic melanoma? In any setting, treatment selection criteria, in order for physicians to choose between immunotherapy plus INT and established immune checkpoint combinations, should also be discussed [93].

Furthermore, INT promises to optimize the quality and intensity of the hosts’ immune system to detect and destroy cancer. Regardless, neoantigens keep changing as a result of endless mutations taking place within the cancer genome; thus, maybe a neoantigen sequence encoded by a vaccine, based on a certain tumor specimen, may not be able to represent the whole genetic evolution of cancer within the human body [94,95]. Malignant cells may constantly alter their epitopes, escaping the immune system of the host, no matter the antigen-specific responses imposed by the vaccine [96].

Furthermore, although neoantigens can now be recognized by NGS technology and potent computational systems, there is no consensus on how neoantigen selection should be performed, or on standardized tools serving this purpose [97,98]. For example, it seems that the grade of immunogenicity of a certain neoantigen depends on the kind of underlying mutation [97,98,99]. In a study of patients with solid tumors characterized by microsatellite instability, it was found that neoantigens resulting from frameshift mutations were widely shared among different tumor clones, and also could bind onto the MCH proteins most frequently expressed by the host [99]. Nonetheless, all kinds of genetic alterations, such as gene fusions, missense mutations, and epigenetics, may produce neoantigens of high immunogenic potential [100]. In this context, innovative tools and predictive models are now required in order to perform proper selection of neoantigens for vaccine construction. Large, comprehensive databases containing thousands of neoantigens of proven immunogenic potential, through in vitro assessments of interaction with T lymphocytes, have already become available [100]. Meanwhile, machine learning applications may save valuable resources by allowing in silico assessment of neoantigens’ ability to interact with T lymphocyte receptors, without performing actual in vitro experiments [101]. It is only possible that neoantigen selection strategies are going to become more concrete and insightful, producing highly effective, specialized vaccines able to counterbalance the immune escape pathways of cancer cells [101,102].

As described above, the development of universal mRNA vaccines may provide an effective alternative to individualized neoantigen vaccines (see Table 3). The phase 2 trial BNT111-01 (NCT04526899) [65] aims to assess the effect of a universal mRNA vaccine, BNT111, which encodes for four pre-specified proteins, NY-ESO-1, MAGE-A3, tyrosinase, and TPTE, in patients with unresectable melanoma. Similarly to individualized vaccine trials, patients receive BNT111 together with an immune checkpoint inhibitor, in this case, cemiplimab. According to the latest announcements, results are encouraging, with more objective responses occurring under BNT111-01 vaccination [65]. Whether mRNA vaccines against melanoma, let alone at all, should encode for common tumor-associated proteins, or patient/cancer-unique personal neoantigens, may rise as a question to be answered in the prospective trials in the future.

Financing production and large-scale distribution of mRNA vaccines, especially ones encoding for individualized neoantigens, is another important concern. Even mRNA vaccines against COVID-19, intended to protect the global population against a pandemic, were not always accessible to residents in middle- and lower-income countries [103,104]. Manufacturing mRNA anti-cancer vaccines, let alone individualized ones, producing one unique medication per patient, requires state-of-the-art technology, including NGS, self-amplifying mRNA platforms, lipid nanoparticle carriers, computational systems, machine learning, and artificial intelligence [31,105]. In this context, it could be argued that such elaborate treatment strategies have a high cost of production, which could not easily be met by lower-income countries.

In addition, producing a totally individualized medication, unique to each patient and their neoplasm, all while being a tremendous scientific achievement, means that only small batches can be produced at a time [105,106]. Nevertheless, more patients may have access to personalized vaccines within the context of clinical trials, without increasing the public health budget of the involved countries. In addition, mRNA vaccines, either universal or individualized, have the advantage of being stored safely and transferred over long distances, allowing central production in specialized centers and distribution to all patients who are to benefit. International regulatory agencies and production companies are now expected to promptly face the responsibility of reaching a consensus, aiming to facilitate the induction of mRNA anti-cancer vaccines into clinical practice, provided that enough evidence supporting their effectiveness and safety has been gathered [31,105,106].

## 9. Critical Appraisal of Clinical Trials Involving mRNA Vaccines

As clinical trials are progressing, clinical outcomes regarding mRNA vaccines are becoming more abundant. Nonetheless, it is still early to assess the effectiveness, safety, and impact of mRNA vaccines. For example, it cannot be doubted that the Keynote-942 findings are of extremely significant importance; however, it draws its assumptions from a small patient sample of 157 patients [34,35,36]. More mature results, as well as outcomes from the larger-scale V940-001 trial, are required to reassure the effectiveness of the mRNA-4157 vaccine. Additionally, no benefit in overall survival has been demonstrated yet for any of the vaccines, as more long-term data are required for this purpose. In fact, even the DMFS benefit in the vaccine scale of the Keynote-942 trial becomes apparent only after the first 18 months since treatment initiation, possibly implying that the vaccine addition does not offer any additional protection from distant disease recurrence early in the course of the disease [34,35,36].

Another concern is about the initial large-scale testing of the individualized mRNA vaccines in the adjuvant setting, where especially prolonged follow up is necessary in order to assess treatment benefit. Also, it could be argued that, when employed in the adjuvant setting, any novel treatment agent cannot prove its actual activity against neoplastic cells, as no objective responses can be seen. The ability of the mRNA-4157 vaccine to induce objective tumor shrinkage is to be assessed in the V940-012 trial [38], recruiting metastatic melanoma patients. The universal BNT111 vaccine started to be administered in the metastatic setting and has already shown responses, although no specific response rate is mentioned in the study report releases to date [65].

Last but not least, employing mRNA-4157 in the adjuvant setting makes it impossible to use a single-agent vaccine arm, as it would be unethical for the patients not to receive the approved immunotherapy [34,35,36]. In this way, the activity of the vaccine is only assessed as part of the combination with the immune checkpoint inhibitor pembrolizumab. Once again, BNT111 may be studied in the context of a better equilibrated clinical trial (BNT111-01), as there is a comparative arm of vaccine monotherapy administered to metastatic, pretreated melanoma patients [65].

## 10. Conclusions

mRNA vaccines, encoding for individual, tumor- and patient-specific neoantigens, are already revolutionizing melanoma oncology. More studies are needed to thoroughly investigate actual patient benefit, as well as to dissect their mechanism of action, in order to and optimize their performance. Nonetheless, they represent a cutting-edge treatment, which has undeniably set a pathway for a perfectly tailored cancer treatment, eventually combining scientific and technological advances of several past decades.

## Figures and Tables

**Table 1:** Active studies involving mRNA-4157 vaccine.

Study	Ref.No	Neoplasms	Phase	Setting	Study Regimen
KEYNOTE-603	NCT03313778	Miscellaneous	I	Systematic	Pembrolizumab plus mRNA-4157
KEYNOTE-942	NCT03897881	Melanoma, resected	IIb	Adjuvant	Pembrolizumab with or without mRNA-4157
V940-001	NCT05933577	Melanoma, resected	III	Adjuvant	Pembrolizumab with or without mRNA-4157
V940-012/INTerpath-012	NCT06961006	Melanoma, unresectable	III	1st line, systematic	Pembrolizumab with or without mRNA-4157
V940-002/INTerpath-002	NCT06077760	Non-small Cell Lung Cancer, resected, st.II-IIIB	III	adjuvant	Pembrolizumab with or without mRNA-4157
V940-009/INTerpath-009	NCT06623422	Non-small Cell Lung Cancer, resectable, not achieving pCR after preoperative platinum-based treatment	III	adjuvant	Pembrolizumab with or without mRNA-4157
V940-004/INTerpath-004	NCT06307431	Renal Cell Carcinoma, Resected	II	adjuvant	Pembrolizumab with or without mRNA-4157
V940-007	NCT06295809	Cutaneous Squamous Cell Carcinoma, resectable	II/III	(Neo)adjuvant	Pembrolizumab with or without mRNA-4157
V940-011/INTerpath-011	NCT06833073	Bladder Cancer, high-risk, non-muscle invasive, endoscopically resected	II	adjuvant	Intravesical BCG with or without mRNA-4157

BCG: Bacillus Calmette–Guérin vaccine, pCR: pathologoanatomic complete response.

**Table 2:** Summary of personalized treatment approaches in melanoma.

Treatment	Mechanism of Action	Advantage	Disadvantage
Peptide vaccines	Peptides are injected into the host and activate APCs	Easy production	Modest clinical efficacy
Dendritic cell vaccines	Monocytes of the host are incubated ex vivo with tumor antigens and/or tumor tissue pieces, under the influence of growth factors and cytokines. The activated DCs produced are injected into the patient	Enhancement of the antigen-presenting capacity of the host Easy subcutaneous administration Have shown efficacy in the context of clinical trials	Modest clinical efficacy Difficult to produce The patient has to be treated in a center with the required facilities
mRNA-transfected DC vaccines	DCs are transfected ex vivo with mRNA encoding for tumor-associated antigens	DCs are able to produce tumor antigens Already established tumor-associated antigen sequences may serve as the template	Antigens encoded not unique to patient and tumor Challenging production chain Need for specialized hospital
mRNA vaccine encapsulated in lipid nanoparticle, not personalized (e.g., BNT111)	mRNA encoding for NY-ESO-1, MAGE-A3, tyrosinase, and TPTE in lipoplex formulation are injected into the host; DCs absorb mRNA and produce the four tumor-associated antigens and provoke immune reaction	Easy to transfer and administer Maybe more affordable Over 90% of patients with cutaneous melanomas express at least one of these four antigens	Not unique to every patient Lack of clinical data
mRNA vaccine encapsulated in lipid nanoparticle, individualized (e.g., mRNA-4157/V940)	Tumor and patient-specific, immunogenic neoantigen loci are identified, mRNA encoding for them is administered to the host, where it is absorbed by DCs, which then produce the targeted neoantigens, exposing them to immune cells.	Unique to every patient Makes use of tumor and host-specific neoantigens Immunogenicity of selected neoantigens may be predicted by in silico studies Easy to transfer, administer	State-of-the-art technology is required for production 1 drug per patient, cannot administer it to large groups High cost

APC: Antigen Presenting Cell, DC: Dendritic Cell.

**Table 3:** Comparison of individualized and universal mRNA cancer vaccine.

mRNA Vaccine	mRNA-4157	BNT111
Encodes for	Up to thirty-four, individual, host, and tumor-specific neoantigens	Four melanoma associated peptides: NY-ESO-1, MAGE-A3, tyrosinase, TPTE
Vector	LNP	LNP
Combined with	pembrolizumab	cemiplimab
Significant trial	Keynote-942 (NCT03897881)	BNT111-01 (NCT04526899)
Available clinical data	Reduces recurrence of high-risk melanoma, combined with pembrolizumab in the adjuvant setting	Improves ORR in unresectable melanoma patients, compared to cemiplimab monotherapy
Potentially applicable to	Melanoma, NSCLC, bladder cancer, RCC	melanoma
advantage	Highly individualized treatment	May benefit larger groups of patients
Disadvantage	Not proven to have a benefit over universal mRNA vaccines Expensive to produce	Not patient-specific Not effective in tumors not expressing the specific encoded antigens

LNP: Lipid Nanoparticles, RCC: Renal cell carcinoma, ORR: Objective Response Rate.

## Data Availability

No new data were created.
